# Clinically impactful metabolic subtypes of pancreatic ductal adenocarcinoma (PDAC)

**DOI:** 10.3389/fgene.2023.1282824

**Published:** 2023-10-30

**Authors:** Jannat Pervin, Mohammad Asad, Shaolong Cao, Gun Ho Jang, Nikta Feizi, Benjamin Haibe-Kains, Joanna M. Karasinska, Grainne M. O’Kane, Steven Gallinger, David F. Schaeffer, Daniel J. Renouf, George Zogopoulos, Oliver F. Bathe

**Affiliations:** ^1^ Department of Oncology, Cumming School of Medicine, University of Calgary, Calgary, AB, Canada; ^2^ Department of Biochemistry and Molecular Biology, University of Calgary, Calgary, AB, Canada; ^3^ Department of Bioinformatics and Computational Biology, University of Texas MD Anderson Cancer Centre, Houston, TX, United States; ^4^ Ontario Institute for Cancer Research, Toronto, ON, Canada; ^5^ Princess Margaret Cancer Centre, University Health Network, Toronto, ON, Canada; ^6^ Pancreas Centre BC, Vancouver, BC, Canada; ^7^ University Health Network, University of Toronto, Toronto, ON, Canada; ^8^ Department of Pathology and Laboratory Medicine, University of British Columbia, Vancouver, BC, Canada; ^9^ Department of Medicine, University of British Columbia, Vancouver, BC, Canada; ^10^ Department of Surgery, McGill University Health Centre, McGill University, Montreal, QC, Canada; ^11^ Department of Surgery, Cumming School of Medicine, University of Calgary, Calgary, AB, Canada

**Keywords:** pancreatic ductal adenocarcinoma, pancreatic cancer, metabolism, deconvolution, prognosis

## Abstract

**Background:** Pancreatic ductal adenocarcinoma (PDAC) is a lethal disease characterized by a diverse tumor microenvironment. The heterogeneous cellular composition of PDAC makes it challenging to study molecular features of tumor cells using extracts from bulk tumor. The metabolic features in tumor cells from clinical samples are poorly understood, and their impact on clinical outcomes are unknown. Our objective was to identify the metabolic features in the tumor compartment that are most clinically impactful.

**Methods:** A computational deconvolution approach using the DeMixT algorithm was applied to bulk RNASeq data from The Cancer Genome Atlas to determine the proportion of each gene’s expression that was attributable to the tumor compartment. A machine learning algorithm designed to identify features most closely associated with survival outcomes was used to identify the most clinically impactful metabolic genes.

**Results:** Two metabolic subtypes (M1 and M2) were identified, based on the pattern of expression of the 26 most important metabolic genes. The M2 phenotype had a significantly worse survival, which was replicated in three external PDAC cohorts. This PDAC subtype was characterized by net glycogen catabolism, accelerated glycolysis, and increased proliferation and cellular migration. Single cell data demonstrated substantial intercellular heterogeneity in the metabolic features that typified this aggressive phenotype.

**Conclusion:** By focusing on features within the tumor compartment, two novel and clinically impactful metabolic subtypes of PDAC were identified. Our study emphasizes the challenges of defining tumor phenotypes in the face of the significant intratumoral heterogeneity that typifies PDAC. Further studies are required to understand the microenvironmental factors that drive the appearance of the metabolic features characteristic of the aggressive M2 PDAC phenotype.

## Introduction

Pancreatic adenocarcinoma (PDAC) is highly lethal, with a 5 -years survival <5% in unresectable cases. Even after curative intent resection, tumor recurs in the majority of instances. While there have been advances in systemic therapies for PDAC, only a small impact on clinical outcomes has been realized. To better understand PDAC biology, several groups have described molecular subgroups with unique clinical and biological features (Collisson et al., 2011; Moffitt et al., 2015; Bailey et al., 2016; Raphael and Cancer Genome Atlas Research Network, 2017; Cao et al., 2021). Most of these efforts have employed bulk (whole tumor) analysis (Collisson et al., 2011; Bailey et al., 2016; Raphael and Cancer Genome Atlas Research Network, 2017; Cao et al., 2021). However, PDAC characteristically has a prominent stromal component consisting of diverse cellular populations including fibroblasts, stellate cells, acinar cells, endothelial cells, and immune cells. The situation is further complicated by highly variable stromal features. While this can be technically addressed by single cell analyses, so far series describing single cell analyses have been too small to provide insight on the effects of individual cellular components on clinical outcomes (Wang et al., 2021; Fang et al., 2022).

It is well known that PDAC afflicts the host with significant metabolic perturbations manifested as impaired glycemic control (Alpertunga et al., 2021; Jeon et al., 2021) and cachexia (Rupert et al., 2021; Zhong et al., 2022). Studies on the circulating metabolome demonstrate distinct features in comparison to controls (Bathe et al., 2011; Mayers et al., 2014), although so far metabolomic subtypes based on the circulating metabolome have not been described. Three metabolic subtypes based on metabolomic profiles have been described in cell lines, including glycolytic and lipogenic subtypes and a subtype with reduced proliferative capacity (Daemen et al., 2015). The metabolic subtypes had differential sensitivities to inhibitors of glycolysis and glutaminolysis. Based on these findings, Karasinska et al. (2020) evaluated the association of glycolytic and cholesterogenic gene expression levels on survival. This targeted analysis demonstrated that glycolytic tumors had the shortest median survival, and cholesterogenic PDACs had the longest survival.

While each of these studies has shed light on very important metabolic features, it is unclear what metabolic features specifically in tumor cells are most impactful on the biology and clinical behavior of PDAC. We postulated that subsets of PDAC had metabolic features that were clinically impactful and potentially actionable from a therapeutic perspective. To separate the effects of the tumor cell compartment from the stromal compartment, we employed a computational deconvolution approach. The most clinically impactful metabolic features of PDAC were identified using a proprietary machine learning algorithm (HighLifeR^™^) (Craig et al., 2022) designed to expose features that were most closely associated with survival outcomes.

## Materials and methods

### Patient samples and data acquisition

In this study, PDAC cases annotated by The Cancer Genome Atlas (TCGA) (*N* = 142) were used as discovery cohort. The normalized RSEM RNASeq data were downloaded from firebrowse.org, and related clinical information was obtained from the Genomic Data Commons Data portal (GDC PDAC). To derive gene expression levels in the tumor compartment, bulk RNASeq data from TCGA were submitted to computational deconvolution. A two-compartment model was employed using the R package DeMixT (Wang et al., 2018; Cao et al., 2022). The discovery cohort consisted of 12,635 protein-coding genes, including 1,499 metabolic genes. The metabolic gene list was aggregated from the Reactome Pathway Database Vol 77. Genes belonging to “Metabolism; Id: R-HAS-143072B.10, Species: *Homo sapiens*.” Data on mutations and copy number variations (hg38) were downloaded using the TCGAbiolinks package in R. Previously defined hypoxia scores of PDAC bulk tumors were downloaded from cBioPortal.

To validate the findings from the discovery cohort, HTSeq RNAseq data and clinical information were obtained from CPTAC (using the R package TCGAbiolinks from the GDC portal), ICGC (from the ICGC Data Portal), and from the COMPASS trial (Aung et al., 2018; Zhang et al., 2019; Cao et al., 2021). Each dataset was independently normalized using the median of ratios, and z-scores of all genes were calculated using the scale function in R. A prediction model based on the prognostic metabolic genes was generated using the WEKA software (Witten et al., 2017). The multilayer perceptron (MLP) algorithm, a form of artificial neural network (Murtagh, 1991), demonstrated the highest accuracy on internal validation.

### Identification of prognostic genes using HighLifeR™

HighLifeR™ (Qualisure Diagnostics Inc., Calgary, Canada) facilitates the interrogation of highly dimensional datasets with relatively limited sample size, to identify features that are most closely associated with survival events. HighLifeR™ employs partial Cox regression using predictions from the latent components method introduced by Li and Gui (2004). The algorithm involves the recursive application of Cox proportional hazards estimates, testing a multitude of genes and patient combinations in a supervised machine learning context. It also employs randomization of training samples into “virtual cohorts” for 20 testing cycles, each including roughly 70% of patients, with resampling to minimize outlier effects. The HighLifeR^™^ statistical mechanism includes: a) extensive combinatorial iterations to establish gene prognosis in variable space; b) selection of the most highly ranked genes with respect to their association with survival; c) development of a combined prognostic rating model. Prognostic genes are chosen based on their recurring top-200 ranking and prognostic influence (Wald statistic) exceeding half of the maximum Wald statistic in the training set (>4.7). Broad utilization of randomization (including in sample distribution for testing and validation, and in combination sequences) reduces the chance of identifying a sample set-bound prognostic pattern.

### Identification of metabolic subgroups

Selected prognostic metabolic genes (*N* = 26) identified using HighLifeR™ were submitted to unsupervised analysis to uncover underlying patterns. The k-means clustering algorithm was utilized using the Euclidean distance metric in conjunction with the complete linkage clustering method, with the number of clusters (k) ranging from 2 to 6. Finally, k = 2 clusters were selected to define the metabolic subtypes, denoted as M1 and M2. For visualization, Euclidean distance metric with complete linkage clustering was employed using the ComplexHeatmap package in R. To conduct the survival analysis, Kaplan-Meier plots were generated using GraphPad PRISM version 8.0.

### Mutation and copy number variation analysis

Mutational and copy number variation analysis was carried out using the R package “maftools.” For somatic copy number variations, GISTIC2.0 was used to download the relevant data. A Fisher’s exact test was used to compare mutation frequencies and copy number variations between groups. To account for multiple comparisons, *p*-values were adjusted using the Benjamin-Hochberg method.

### Differentially expressed genes analysis

Differentially expressed genes (DEGs) were identified using the limma-voom function in R (Law et al., 2014). Briefly, data were transformed to log2 counts per million reads (CPM). Genes with less than 1 CPM were excluded from the analysis of differentially expressed genes. The metabolic subtype with better survival (M1) was used as the reference group. We adjusted for multiple comparisons using the Benjamini-Hochberg method. The significance threshold for this analysis was an adjusted *p*-value of ≤0.05 and log fold changes of ≥±1. Ultimately, 551 DEGs were identified based on these criteria.

### Gene set enrichment and pathway analysis

Gene set enrichment analysis (GSEA) was performed using version 4.1.0 of the Broad Institute GSEA software. Using all protein coding genes, the 50 Hallmarks version 7.5 gene sets from the Broad Institute were interrogated. Then a focused evaluation for enrichment of metabolism-related pathways was performed, limiting the input to the 1,499 metabolic genes present in the expression dataset from the discovery cohort. The 46 pathways dedicated to metabolism were sourced from the Reactome database (data available at https://reactome.org/PathwayBrowser/#/R-HSA-1430728). We determined significance levels through a permutation-based approach and adjusted for multiple testing using the Benjamini-Hochberg method to calculate the false discovery rate (FDR). The enrichment score was computed using a ranking-based metric, which measures the cumulative distribution of genes within a gene set relative to the entire dataset. Results with a significance cutoff of *p* < 0.05 and an FDR threshold of <25% were considered statistically significant.

For the IPA (Ingenuity Pathway Analysis) analysis, a total of 551 DEGs, along with their adjusted *p*-values and fold changes, were used as input. The IPA analysis was conducted using the Qiagen IPA platform (https://digitalinsights.qiagen.com/products-overview/discovery-insights-portfolio/analysis-and-visualization/qiagen-ipa/). Fisher’s exact test was utilized to assess the statistical significance of pathway and function enrichment. We controlled for multiple testing using the Benjamini-Hochberg correction and calculated activated z-scores to predict the likely activation state of biological processes based on DEGs. A significance cut-off of nominal *p*-value 0.05 was applied for the canonical pathways, while a Benjamini-Hochberg (BH) *p*-value of 0.05 was used for the functional analysis. Pathway visualizations were generated using various R packages.

### Single cell analysis

To explore single-cell characteristics of PDAC primary tumors and metastasis samples, we retrieved the NCBI-GEO dataset with the ID GSE154778 (Lin, W. et al., 2020). The dataset included single cell RNASeq data from 10 primary PDACs and 6 samples from metastases. The RNA-seq data for each sample were obtained following the methodology described in the study. The focus of the downstream analysis was on epithelial tumor cells (ETCs) to validate the presence of inter-tumor heterogeneity. Classification of ETCs was based on marker genes, including EpCAM and KRT19. Log-normalized data and z-scores for prognostic metabolic genes were calculated for each tumor sample.

### Drug sensitivity testing

Pharmacogenomic profiles from drug screening studies of PDAC cell lines from CCLE and gCSI were downloaded from Orcestra.ca (https://orcestra.ca/pset). Using the MLP model, cell lines were classified as M1 or M2. The data from CCLE and gCSI were not combined to mitigate potential confounding factors such as variations in drug concentration range or cell viability assay methods. To compare the drug sensitivity status between the two subtypes, the Wilcoxon signed-rank test was used to compare the distribution of the area above the dose-response curve (AAC) values per drug across the subtypes. *p*-values were adjusted using the Benjamini-Hochberg method to account for multiple testing.

### Additional statistical details

In addition to the analyses described above, continuous variables were compared using the Student’s t-test, and the Mann-Whitney U test was used for non-parametric data. Proportions were compared using the chi-square test. Log-rank tests were used to compare survivals between groups. Statistical analyses were executed using RStudio, GraphPad PRISM version 8.0, and SPSS version 26.

## Results

### Identification of two prognostic metabolic phenotypes

We used data from The Cancer Genome Atlas (TCGA) PDAC cohort consisting of 142 patients as our discovery dataset. The majority of the TCGA PDAC samples were resected, and RNASeq was performed in bulk samples from primary tumors. The DeMixT algorithm was applied to determine the expression level of each gene that was attributable to the tumor compartment. HighLifeR^™^, a proprietary machine-learning algorithm designed to identify features that most strongly contribute to survival outcomes, was applied to the deconvolved tumor-specific expression of 1,499 genes with a known metabolic function. Briefly, HighLifeR^™^ calculates the Cox proportional hazards in an exhaustive series of permutations consisting of combinations of genes and patients. A set of 26 genes was identified as most consistently and significantly associated with overall survival (OS). Genes had diverse metabolic functions, including roles in carbohydrate, amino acid, and lipid metabolism (Supplementary Table S1). Interestingly, dihydropyrimidine dehydrogenase (DPYD), the rate-limiting enzyme responsible for the metabolism of 5-fluorouracil, emerged in the list of prognostic metabolic genes. Tumor expression of DPYD has been linked to resistance to fluoropyrimidines (Gustavsson et al., 2009; Zhang et al., 2017). Upon searching the Human Protein Atlas, six of these genes (SPTLC3, PYGL, GMPS, ACSF3, SLC16A1, and PGM1) were confirmed to be prognostic in PDAC, based on expression at the protein level.

**FIGURE 1:**
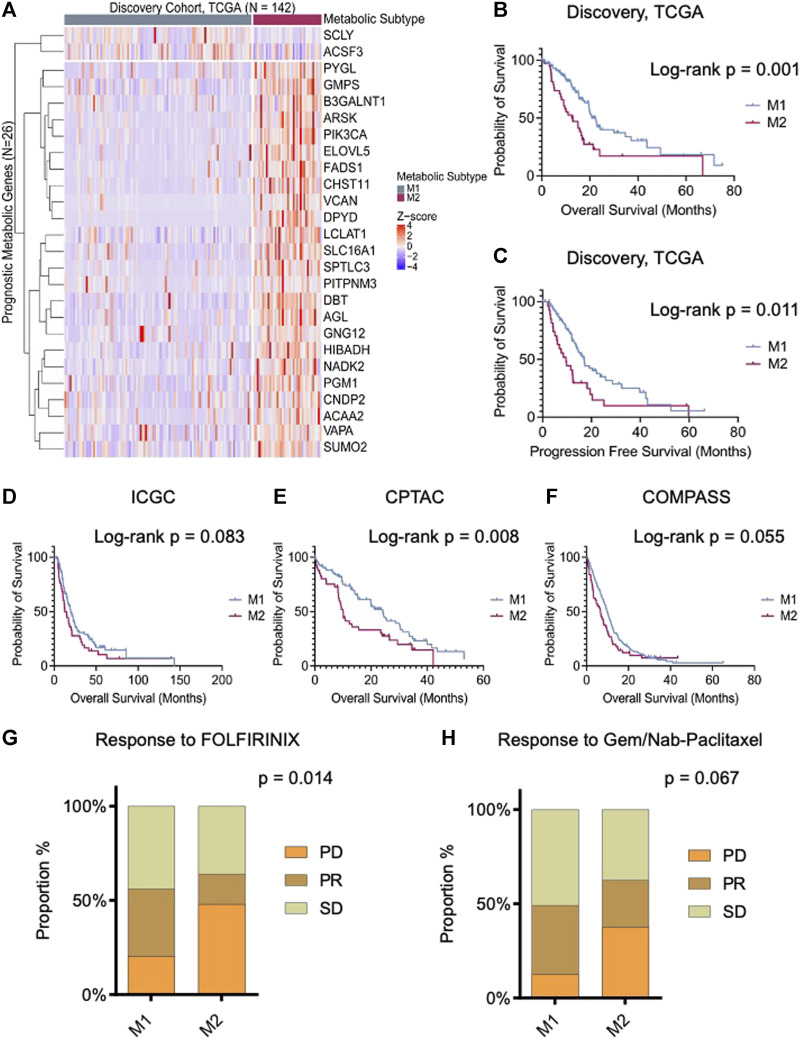
Prognostic tumor metabolic subtypes in pancreatic ductal adenocarcinoma (PDAC). **(A)** Unsupervised clustering (k = 2) illustrating distinct metabolic subgroups in the TCGA PDAC cohort (*N* = 142). The heatmap depicts expression levels of prognostic metabolic genes within the identified subtypes, highlighting both “lower-risk” (M1) and “high-risk” (M2) groups. **(B,C)** Kaplan-Meier plot displaying the overall and progression-free survival of the metabolic subtypes in the discovery cohort, demonstrating significant differences in survival outcomes. **(D−F)** Overall survival analysis of resectable PDAC patients: International Cancer Genome Consortium (ICGC, *n* = 172), Clinical Proteomic Tumor Analysis Consortium (CPTAC, *n* = 140), and unresectable patients from the COMPASS cohort (*n* = 272) stratified according to predicted metabolic subtypes. **(G,H)** Bar chart presents the responses to the first line treatment in COMPASS cohort. M1 subtype exhibits stable and partial responses to both FOLFIRINOX and Gemcitabine/Nab-paclitaxel. On the contrary, M2 group shows poor response to gemcitabine and combined therapy. Log-rank and Chi-square *p*-values are shown in the figure.

An unsupervised analysis focused on the 26 prognostic genes revealed a pattern of co-expression. Two clusters were apparent (M1 and M2; Figure 1A). In the largest subgroup (M1), which consisted of 104 patients (74.2%), most genes were relatively downregulated. In the M2 subgroup, which comprised 38 patients (26.8%), the prognostic genes were frequently overexpressed, with the exception of SCLY and ACSF3. This latter subgroup had a significantly truncated overall survival (OS) in comparison to the M1 subtype (log-rank *p*-value 0.001; Figure 1B). Similarly, we observed a shorter progression-free survival (PFS) in the M2 subtype (*p* = 0.011; Figure 1C). Interestingly, the clinical features in the two metabolic subtypes were indistinguishable, including features that were known to influence survival (Table 1). Information on diabetes status was available for 122 patients. In patients with the M1 phenotype, 25 of 86 (29.1%) patients had diabetes. In the M2 phenotype, 7 of 34 (20.5%) patients had history of diabetes. This was not significantly different.

**TABLE 1:** Patient characteristics in TCGA cases with each metabolic subtype phenotype.

Characteristics	PDAC tumor metabolic subtypes		*p-value*
	M1 (Lower-risk) No (*n* = 104) (%)	M2 (High-risk) No (*n* = 38) (%)	
Age (years)			0.440
Mean ± SD	64.54 ± 10.633	66.58 ± 10.892	
Sex			0.684
Female	48 (46.2)	19 (50.0)	
Male	56 (53.8)	19 (50.0)	
Race			0.761
Asian	5 (5.0)	3 (7.9)	
African American	4 (4.0)	1 (2.6)	
White	91 (91.0)	34 (89.5)	
Histologic Grade			0.219
G1	16 (15.4)	4 (10.5)	
G2	60 (57.7)	19 (50.0)	
G3	28 (26.9)	14 (36.8)	
G4	0 (0.0)	1 (2.6)	
Tumour Stage			0.717
T1	2 (1.9)	1 (2.7)	
T2	13 (12.5)	3 (8.1)	
T3	87 (83.7)	33 (89.2)	
T4	2 (1.9)	0 (0.0)	
Lymph Node Stage			0.459
N0	28 (27.2)	8 (21.1)	
N1	75 (72.8)	30 (78.9)	
Metastatic Stage			0.871
M0	47 (94.0)	19 (95.0)	
M1	3 (6.0)	1 (5.0)	
Smoking History Category			0.538
1	33 (38.8)	17 (51.5)	
2	12 (14.1)	5 (15.2)	
3	21 (24.7)	4 (12.1)	
4	14 (16.5)	6 (18.2)	
5	5 (5.9)	1 (3.0)	
Alcohol History			0.830
No	37 (38.1)	13 (36.1)	
Yes	60 (61.9)	23 (63.9)	
Diabetes History			0.270
No	61 (70.9)	29 (80.6)	
Yes	25 (29.1)	7 (19.4)	

A predictive model based on the multilayer perceptron (MLP) classifier was generated for validation studies. The accuracy of this model on internal validation was 95.8% ± 0.8%. RNASeq data were acquired from three different cohorts to validate our findings. These included the ICGC study (*N* = 172), an analysis of tumor cells enriched by laser capture microdissection (LCM) from surgical patients; the COMPASS trial (*N* = 272) (Aung et al., 2018), LCM-enriched tumor cells from unresectable PDAC (Zhang et al., 2019); and the CPTAC study (*N* = 140), a bulk analysis of mostly surgical patients (Cao et al., 2021). The MLP classifier was based on gene expression z-scores, facilitating the classification of metabolic phenotype, including in bulk transcriptomes. The proportions of each cohort that consisted of the M2 phenotype were approximately the same in each cohort: 23.8% in the ICGC cohort; 23.2% in the COMPASS cohort; and 30.0% in the CPTAC cohort (Supplementary Figure S1). However, in the COMPASS study, a larger proportion of patients with documented metastatic PDAC (54 of 219; 24.7%) were M2 in comparison with the fraction of locally advanced cases that were M2 (2 of 28; 7.1%) (*p* = 0.01).

In each validation cohort, cases with the M2 phenotype had the shortest overall survival (Figures 1D–F). In cases from the COMPASS trial that consisted of patients on palliative chemotherapy, the metabolic subtype also conferred differences in chemotherapy response rates. Specifically, following treatment with FOLFIRINOX (FFX), disease progression was more commonly reported in M2 tumors (48.0% vs. 20.4%; *p* = 0.014), and response rate was higher in M1 tumors. Similarly, progressive disease (PD) was more common in patients with M2 tumors treated with gemcitabine/nab-paclitaxel (37.5% vs. 12.7%; *p* = 0.067) (Figures 1G, H).

### Metabolic subtypes and PDAC molecular subtypes

**FIGURE 2:**
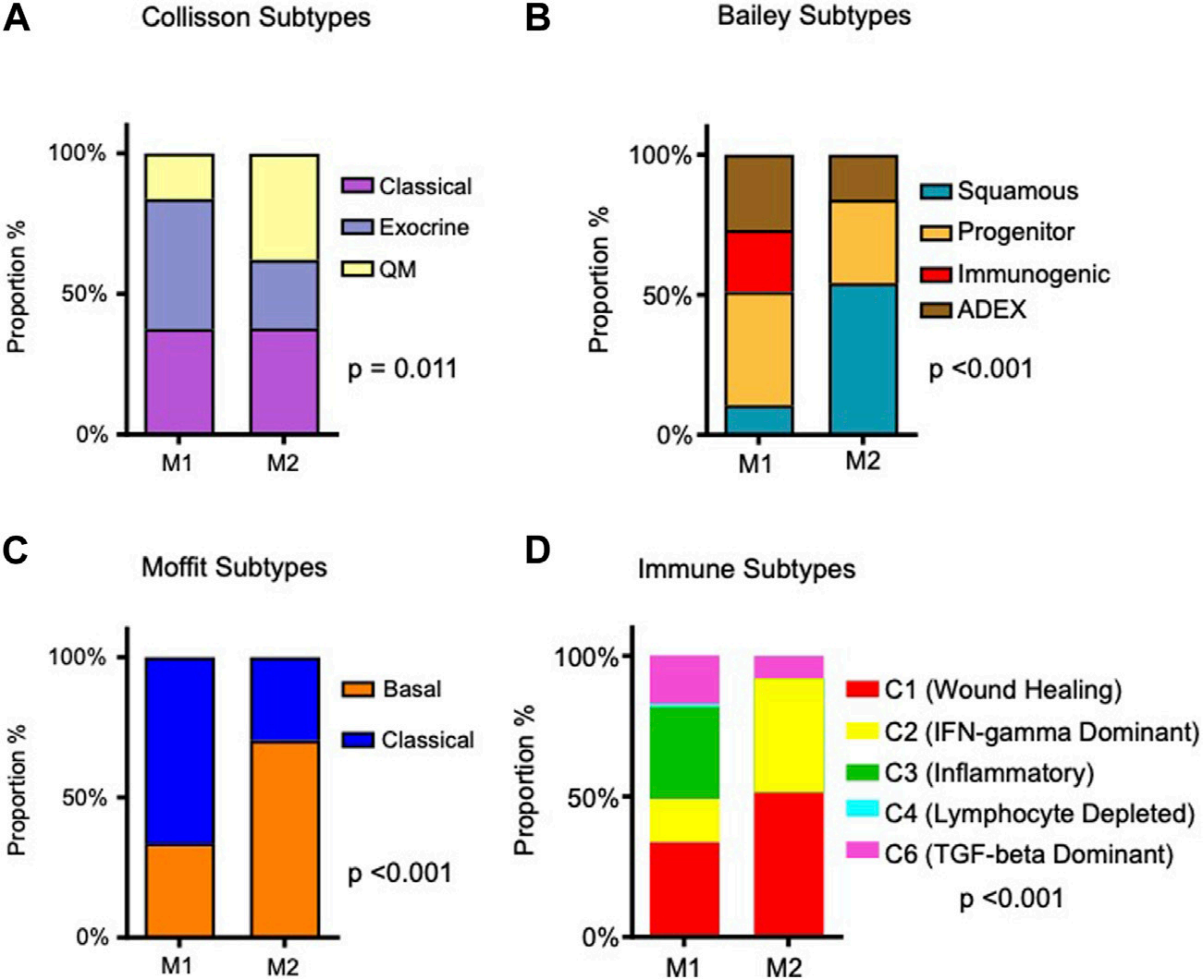
Association of identified metabolic subtypes with PDAC molecular subtypes and immune subtypes. **(A)** Stacked bar charts depicting the distribution of patients in the M1 and M2 PDAC tumor metabolic subtypes, alongside their intersection with previously reported PDAC molecular subtypes (Collison et al., 2011; Moffit et al., 2015; Bailey et al., 2016). **(B)** Proportions of immune subtypes as described by Thorsson et al. (2018) are presented within the identified tumor metabolic subtypes. Chi-square *p*-values are provided to assess the significance of associations.

The metabolic subtypes were evaluated in the context of molecular subtypes described by Collisson et al., 2011; Moffitt et al., 2015; Bailey et al., 2016. Of note, the Moffit subtypes were also derived from a computational deconvolution method. Using this approach, two tumor phenotypes (classical and basal-like) were identified, and the phenotype of the stromal compartment could also be dichotomized (normal and activated), each with prognostic significance. The relationship of other molecular subtypes with our metabolic subtypes is illustrated in Figures 2A–C. In each case, there were significant differences between M1 and M2 tumors. M2 tumors (with the worst prognosis) were most frequently Moffit basal-like. In comparison to the M1 subtype, M2 tumors were more frequently Collisson quasimesenchymal and Bailey squamous. The Moffit basal-like, Collisson quasimesenchymal and Bailey squamous subtypes reportedly have the worst prognosis.

Recently, using pan-cancer data from 33 diverse tumor types, Thorsson et al. identified six immune subtypes based on the composition of the immune infiltrate and expression of immunomodulatory genes (Thorsson et al., 2018). Immuno-inflammatory patterns differed significantly in M1 and M2 tumors (*p*-value < 0.001; Figure 2D). Specifically, M1 tumors were comprised of more inflammatory (C3) and TGF-beta dominant (C6) immune subtypes; M2 tumors were almost all wound healing (C1) and IFN-gamma dominant (C2) subtypes, although 8.1% were also TGF-beta dominant subtype. C1 tumors have increased expression of angiogenic genes and a Th2 cell bias in their adaptive immune cell infiltrate. C2 tumors have a strong CD8 signal, but high T cell receptor diversity. In the pan-cancer paper by Thorsson et al., C1 tumors and C2 tumors had a worse prognosis than C3 tumors, and C4 and C6 tumors had the worst survival outcomes.

### Biological features of metabolic subtypes

There were no significant differences between M1 and M2 tumors in mutation frequency or copy number variations. To gain an understanding of the biological features that characterized our two metabolic subtypes, we first performed a gene set enrichment analysis (GSEA), focusing first on the 50 hallmark gene sets in MSigDB. Using deconvolved gene expression levels from the tumor compartment from the TCGA dataset, pathways with the most significant enrichment in the poor prognosis M2 subtype included protein secretion, UV response, mammalian target of rapamycin 1 (mTORC1) signaling, and heme metabolism (Table 2). Metabolic pathways that did not quite reach significance included glycolysis, androgen response, angiogenesis and adipogenesis. A similar analysis was performed on the two datasets derived from physically enriched tumor cells. In the ICGC dataset, protein secretion, oxidative phosphorylation, MYC targets and DNA repair were enriched. In the COMPASS dataset, protein secretion and MYC targets were enriched. Finally, using bulk data from CPTAC, protein secretion, MYC targets and DNA repair pathways were enriched.

**TABLE 2:** Gene set enrichment analysis in M1 and M2 subtype in the TCGA cohort (the discovery cohort) using the Broad Institute 50 Hallmarks gene sets.

Enriched Hallmark functions	Normalized enrichment score (NES)	Nominal *p*-value	FDR (q-value)
Protein secretion*	1.85	0.000	0.214
UV response*	1.62	0.015	0.228
mTORC1 signaling*	1.67	0.022	0.211
Heme metabolism*	1.52	0.039	0.228
Unfolded protein response	1.69	0.020	0.383
DNA repair	1.56	0.022	0.282
MYC targets	1.68	0.035	0.259
Glycolysis	1.45	0.069	0.232
Androgen response	1.47	0.071	0.231
Angiogenesis	1.47	0.083	0.250
Adipogenesis	1.44	0.087	0.229
Peroxisome	1.39	0.097	0.253
G2M checkpoint	1.55	0.105	0.228
E2F targets	1.52	0.127	0.209

* = Significant based on both nominal *p*-value (≤0.05) and FDR (≤0.25).

**FIGURE 3:**
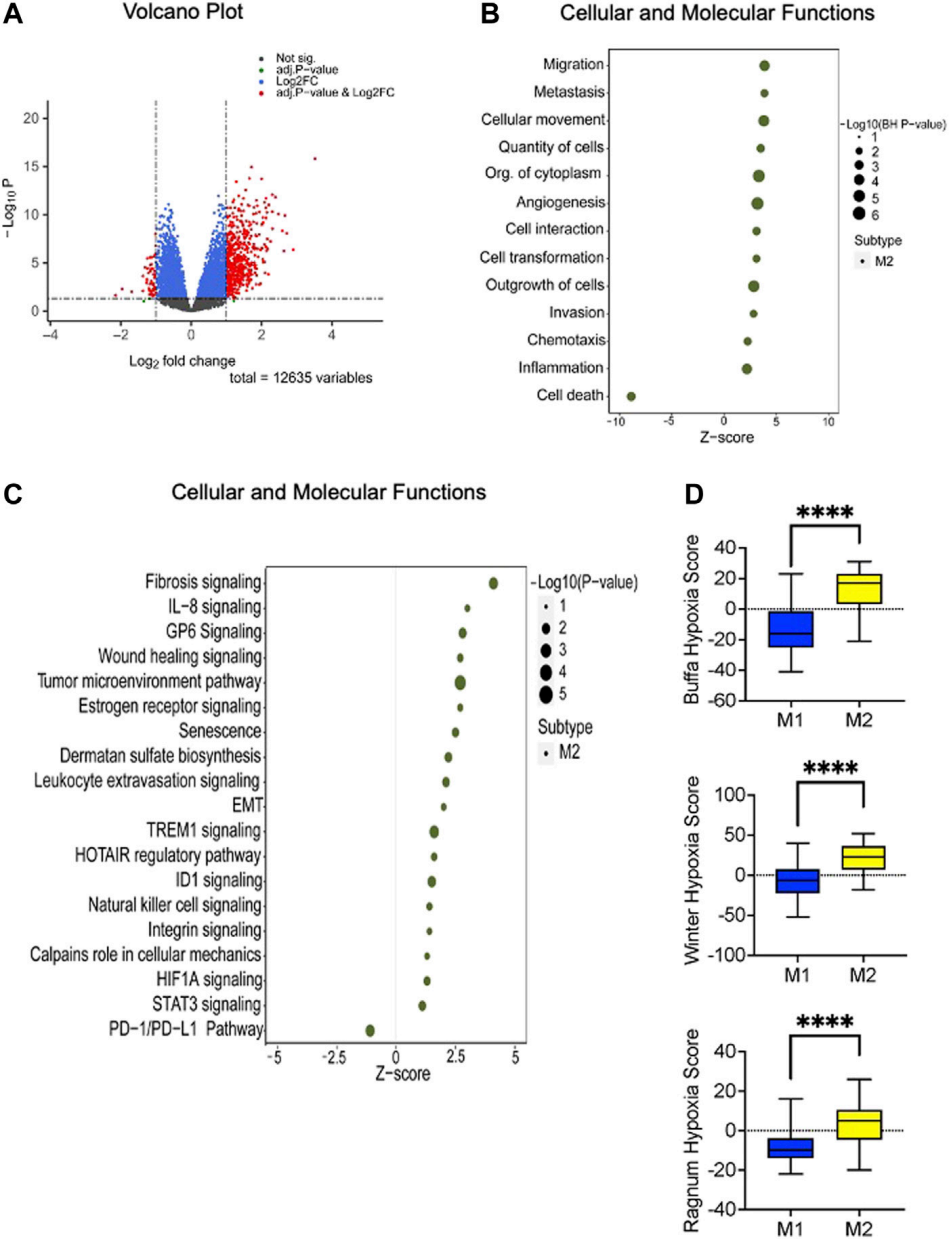
Altered canonical pathways and functions linked to the high-risk (M2) subtype. **(A)** Volcano plot demonstrating the differentially expressed genes (*n* = 551) between the identified metabolic subtypes. Statistical significance is represented by BH adjusted *p*-value of 0.05 and a Log2 fold change threshold of ±1. **(B)** Ingenuity Pathway Analysis (IPA) of the differentially expressed genes reveals that the high-risk subtype is positively enriched in cellular proliferation, migration, and invasion. The enrichment analysis yields adjusted *p*-value less than or equal to 0.05. **(C)** Disrupted canonical pathways associated with M2 metabolic subtype encompass crucial signaling pathways. Fisher’s exact test results indicate a significance level of less than or equal to 0.05. The x-axis represents the activated z-score with sizes indicating the level of significance (right side of the plot). **(D)** The box plot illustrates the hypoxia scores associated with the identified metabolic subtypes. Based on previous studies (Winter et al., 2007; Buffa et al., 2010; Ragnum at el., 2014) patients with the M2 phenotype exhibits higher scores than M1 phenotype. The statistical significance of these comparisons was assessed using the Mann-Whitney U test, with a *p*-value threshold set at 0.05 or less.

To derive a deeper understanding of the potential biological features distinguishing the two metabolic subtypes, we applied a knowledge-based approach using Ingenuity Pathways Analysis (Qiagen, Redwood City, CA). There were 551 differentially expressed genes (adjusted *p*-value 0.05 and Log2fold changes equal or greater than ± 1) that informed the analysis (Figure 3A, Supplementary Data Sheet S1). Top molecular and cellular functions positively enriched in M2 tumors included cell migration, invasion, angiogenesis, proliferation, and inflammation (Figure 3B). Canonical pathways that were significantly enriched included fibrosis signaling, the tumor microenvironment pathway, estrogen receptor signaling, TREM1 signaling, ID1 signaling, STAT3 signaling, and HIF1 signaling (Figure 3C). Interestingly, ID1 expression leads to IL-6 production, activating STAT3 (Meng et al., 2020), which is known to drive tumor associated macrophages toward an immunosuppressive phenotype, also promoting angiogenesis, invasion and epithelial-mesenchymal transition (EMT) (Chang et al., 2013). TREM1 similarly appears to amplify the inflammatory response, including production of IL-6; blockade of TREM1 has reportedly attenuated tumor growth in an experimental PDAC model (Shen and Sigalov, 2017).

To further dissect the metabolic features that characterized our two metabolic subtypes, we performed a GSEA using only genes with a known metabolic function. The high-risk M2 subtype had significant enrichment in glucagon signaling, glycogen metabolism, and regulation of insulin secretion. Other enriched metabolic pathways (but to a lesser degree) included sphingolipid and triglyceride metabolism (Table 3).

**TABLE 3:** Metabolism-focused gene set enrichment analysis of M1 and M2 subtypes.

Enriched metabolic pathways	Normalized enrichment score (NES)	Nominal *p*-value	FDR (q-value)
Glucagon signaling in metabolic regulation*	2.08	0.000	0.002
Glycogen metabolism*	1.79	0.006	0.094
Regulation of insulin secretion*	1.55	0.039	0.251
Metabolism of water-soluble vitamins and cofactors	1.57	0.017	0.267
Sphingolipid	1.59	0.026	0.321
Phase II conjugation of compounds	1.50	0.031	0.292
Triglyceride metabolism	1.46	0.045	0.312

* = Significant based on both nominal *p*-value (≤0.05) and FDR (≤0.25).

Seemingly, the findings related to carbohydrate metabolism were contradictory. Firstly, in M2 PDACs, glucagon signaling, which promotes gluconeogenesis and glycogen synthesis, was enriched. On the other hand, while the enrichment of glycolysis was not statistically significant (nominal *p* = 0.07, FDR 0.23), virtually all of the key genes corresponding to enzymes promoting glycolysis were elevated. Secondly, there was evidence of both glycogen synthesis (NES 1.50, nominal *p* = 0.075, FDR = 0.054) and glycogenolysis (NES 1.74, nominal *p* = 0.009, FDR = 0.013) (Supplementary Figures S2A, B). The rate limiting enzymes involved in both of these processes are both increased in M2, although there was a 2-fold upregulation of PYGL (promoting glycogenolysis); GYS1 (encouraging glycogen synthesis) was increased by less than 10%. Of note, in cell lines in hypoxic conditions, there is an initial rapid increase in glycogen synthesis catalyzed by GYS1 followed by PYGL-dependent glycogen breakdown (Favaro et al., 2012). The expression of GYS1 is HIF1α-dependent, but PYGL is not consistently HIF1α-dependent; PYGL depletion impairs tumor growth, as glycogen breakdown is integral to tumor cell function and growth.

### Metabolic phenotype as a function of hypoxia

To explore the potential role of hypoxia, we compared the hypoxia scores between M1 and M2 PDACs using three previously described methods (Winter et al., 2007; Buffa et al., 2010; Ragnum et al., 2014). In this analysis using bulk transcriptomic data, M2 tumors had significantly higher hypoxia scores (Figure 3D). However, when a focused GSEA on the transcriptome ascribed to the tumor compartment was performed using hypoxia-related gene sets obtained from the Broad Institute MSigDB (Hallmark Hypoxia) and Reactome (Cellular Response to Hypoxia), there was no significant enrichment (nominal *p*-value 0.11). This suggests that any hypoxia in the tumor microenvironment predominantly affects the stromal compartment rather than the tumor compartment. Other studies have similarly suggested that there is considerable intratumor heterogeneity of hypoxia in human and murine PDAC tumors (Dhani et al., 2015; Lee et al., 2016). Moreover, inflammatory cancer-associated fibroblasts (iCAFs) preferentially localize in hypoxic regions (Mello et al., 2022).

Hypoxia is thought to stem from the desmoplastic stroma of PDAC, possibly by contributing to increased intratumoral pressures, compressing tumor cell vasculature (Chauhan et al., 2014). In that same vein, we evaluated related factors in the two metabolic subtypes. Tumor size was considered, as we postulated that larger tumors would be more susceptible to hypoxia. Neither tumor size nor T-stage were significantly different between M1 and M2 tumors. Angiogenesis is a known consequence of hypoxia (Pugh and Ratcliffe, 2003). Indeed, IPA analysis demonstrated enrichment in genes involved with angiogenesis in M2 tumors. Finally, the proportion of tumor that consists of stroma may also reflect the state of tumor oxygenation. Previously, in PDAC, it was reported that HIF-1α activation by tumor hypoxia causes secretion of sonic hedgehog (SHH) by cancer cells, which stimulates deposition of fibrous tissue by stromal fibroblasts (Spivak-Kroizman et al., 2013). However, in contrast to that observation, tumor content was significantly greater in the putatively hypoxic M2 tumors, whether estimated histologically (tumor nuclei count), or by ABSOLUTE or DeMixT (Supplementary Figure S3).

### Upstream regulators associated with the high-risk metabolic subtype

We took a causal analysis approach using the IPA advanced network tool to infer the identity of upstream regulatory molecules and associated mechanisms related to our two metabolic subtypes (Krämer et al., 2013). Based on the upstream regulator analysis using the differentially expressed genes, we identified a number of upstream regulators that were activated in the high-risk metabolic subtype, including AGT (z-score 4.115), TGFβ1 (z-score 3.734), SMARCA4 (z-score 3.66), NFkB (z-score 3.238), STAT1 (z-score 3.106), and VEGF (z-score 3.084). There were also a number of inhibited upstream regulators associated with the M2 phenotype, including IKZF1 (z score −2.82), IKZF3 (z-score −2.442), EWSR1-FLI1 (z-score −2.236), GMNN (z-score −2.236), and TAF4 (z-score −2.186).

Angiotensinogen (AGT), a substrate for angiotensin II, the primary peptide for the renin-angiotensin system (RAS) was predicted to directly control the expression of 43 DEGs in our dataset: 34 of them were activated, and 9 of them were inhibited (overlap *p*-value 6.33E-07, BH *p*-value 2.72E-05). Evidence suggested that stimulation of local RAS induces VEGF expression, promoting PDAC tumor growth (Anandanadesan et al., 2007).

TGFβ1 was associated with 62 upregulated DEGs (overlap *p*-value 4.97E-06, BH *p*-value 6.91E-05), of which 21 overlapped with the AGT network. Angiotensin II (ANG II) increases the expression of TGFβ-1 (Weigert et al., 2002). In PDAC, TGFβ encourages tumor growth by regulating EMT through SMAD4. It has been previously reported in different types of cancer that cellular proliferation, migration, and angiogenesis could also be influenced by TGFβ, partially mediated by VEGF (Li et al., 2005; Ferrari et al., 2009). TGFβ is also a prominent mediator of NFκB activation and PTEN suppression in pancreatic cancer (Chow et al., 2010). SMARCA4 is associated with mitotic spindle, apical junction, and PI3/AKT/mTOR signaling pathways; it is increased in many types of cancer and is reportedly associated with the poor prognosis in other cancers (Peng et al., 2021; Guerrero-Martínez and Reyes, 2018). In all, the aggressive M2 phenotype has a number of potentially causative pathways, some of which may be interrelated.

### Single cell studies

While this study described metabolic phenotypes in the tumor compartment (and therefore differed from bulk transcriptomic studies), in effect the deconvolved gene expression data that comprised the M1 and M2 phenotypes are the product of the average gene expression values in tumor cells. The question remained whether tumor cells existed in a uniform metabolic state within a tumor, or whether there was phenotypical diversity at the single cell level. To explore this, we accessed data from a single cell RNA-Seq studies on 16 tumors (10 primary tumors and 6 metastases) (Lin, W. et al., 2020). Epithelial tumor cells were identified, and expression levels for each of the metabolic genes associated with PDAC prognosis were determined for each individual tumor cell. Samples with at least 100 epithelial tumor cells (ETCs) were used in this analysis. One metastasis sample (MET03) and three primary tumors (P01, P02, and P03) were excluded from analysis because of insufficient numbers of cells. Supplementary Figure S4 shows the degree of heterogeneity within each tumor. For ease of comparison, the gene order on the heatmap is the same as in Figure 1A.

There were several interesting observations. First, there was significant diversity in the expression of prognostic metabolic genes between cells within any individual tumor. Second, the proportion of tumor cells with greater similarity to M1 or M2 phenotype varied little. Third, the genes that were characteristically upregulated in M2 PDACs were not as closely co-related at the single cell level. Finally, there were individual tumors where more of the prognostic metabolic genes were upregulated in more cells. Likely, these types of tumors represented PDACs that would be recognizable as M2 PDACs in the bulk analysis.

### Potential drug targets

We used the PharmacoGx platform to determine some potential drug targets that might particularly target the high-risk M2 subtype. Initially, we obtained two different PDAC cell line datasets including the Cancer Cell line Encyclopedia (CCLE, *n* = 41) (Cancer Cell Line Encyclopedia Consortium, Genomics of Drug Sensitivity in Cancer Consortium, 2015), and the Genetech Cell Line Screening Initiative (gCSI, *n* = 35) (Klijn et al., 2014). The multilayer perceptron classifier was applied to cell lines to classify metabolic phenotype. Of the CCLE PDAC cell lines, 9 (22.0%) had the M2 phenotype; 10 of the gCSI cell lines (28.6%) were M2 (Supplementary Figure S5). In the CCLE collection, there was no differential drug sensitivity between M1 and M2 cell lines. In gCSI cell lines, based on the nominal *p*-value, M2 cell lines were more sensitive to docetaxel and erlotinib (*p* = 0.048 and *p* = 0.019, respectively). However, following correction for multiple comparisons, this was not significant (Supplementary Figure S6).

## Discussion

Metabolic reprogramming is one of the hallmarks of cancer (Hanahan and Weinberg, 2011). As with other hallmarks of cancer, metabolic reprogramming confers a growth advantage to malignant cells. Metabolic derangements are potentially attractive therapeutic targets, especially perturbations that adversely affect patient health and survival. We have identified two novel metabolic subtypes based on metabolic genes most closely associated with survival events. One of these phenotypes (M2) has a significantly worse prognosis. The M2 phenotype has a significant overlap with the poor prognosis molecular subtypes described by Collisson et al., 2011; Moffitt et al., 2015; Bailey et al., 2016. The pattern of immune infiltrate or M2 tumors differs from M1 tumors. In addition to biological features that would encourage tumor growth, M2 PDACs appear to be hypoxic (in the stromal compartment) and have increased glycogen turnover.

Others have described metabolic subtypes of PDAC (Daemen et al., 2015; Peng et al., 2018; Karasinska et al., 2020). The approach taken by others has involved identifying tumors based on selective metabolic pathway genes. Our approach was different. Instead of evaluating known metabolic pathways as a whole, we took an unbiased approach to identify metabolic genes that most consistently associated with survival events. The result was a list of 26 genes with diverse functions; their pattern of expression dictated one of two metabolic phenotypes with distinct biological and clinical features.

One challenge in the study of PDAC is its cellular heterogeneity. PDACs vary considerably in their composition, some containing large numbers of tumor cells and others dominated by desmoplastic stroma. The stromal constituents are similarly diverse. As a result, there are limitations in how one may interpret molecular studies from bulk samples. To address this, we used a computational deconvolution method to determine the proportion of expression of each gene that could be ascribed to the tumor compartment. A simple two-compartment model was applied using DeMixT. There are numerous other approaches to computational deconvolution, many capable of deconvolution to many cell types (Newman et al., 2015; Avila Cobos et al., 2020). Technical and biological biases can affect the accuracy of any deconvolution technique (Vallania et al., 2018). The metabolic subtypes we described in deconvolved data were observable in samples derived from physical enrichment, by laser capture microdissection (LCM). LCM too has limitations: it is virtually impossible to isolate an entirely pure population of tumor cells, and thermal injury to cells may alter any gene expression measurements. However, it was reassuring that the model built from computational deconvolution could be replicated in physically enriched tumor cells.

Even in a pure tumor cell population isolated from a PDAC, it is likely that there is significant heterogeneity in cells from the same tumor. Therefore, the metabolic features attributable to our two metabolic subtypes represent an average of what may be present throughout the entire tumor sample. Indeed, using sc-RNASeq data, we found that there is substantial cell-to-cell diversity in the expression levels of the individual genes within any single PDAC. Further studies will be required using high resolution spatial transcriptomics methods to identify the determinants of intratumoral heterogeneity in biological processes such as metabolism. However, current methods may not have sufficient resolution at the cellular level, and gene representation of any single cell analysis technique is still not comprehensive.

Unlike previous studies (Daemen et al., 2015; Peng et al., 2018), we did not specifically evaluate any drugs targeting metabolic pathways. Rather, we evaluated the clinical and *in vitro* responses to chemotherapies currently in clinical use. In the COMPASS cohort, M1 tumors had a higher rate of PR and SD in response to FOLFIRINOX; a trend toward higher response rate was seen in M1 tumors treated with gemcitabine + paclitaxel. *In vitro*, M2 tumors were slightly more sensitive to docetaxel, another taxane. We view the *in vitro* data as weak, as only one of the cell line collections demonstrated a slight difference in sensitivity, and this could not be replicated in the other cell line collection. Another reason for this discrepancy may be the inclusion of a fluoropyrimidine (5-FU) in FOLFIRINOX and gemcitabine in paclitaxel-gemcitabine; high DPYD expression in M2 tumors confers resistance to fluoropyrimidines and gemcitabine (Gustavsson et al., 2009; Zhang et al., 2017; Tsukahara et al., 2022). Our *in silico* experiments were limited by statistical power. Further studies would be required to explore the utility of our metabolic classification in treatment selection, especially as it pertains to drugs interfering with specific metabolic functions.

Perhaps the most well described feature of cancer cells is their high rate of glycolysis followed by lactic acid fermentation even in normoxic conditions, known as the Warburg effect. One particularly intriguing feature of M2 tumors was the simultaneous upregulation of genes related to glycogen synthesis, glycogenolysis, and glycolysis. Under nutrient deprivation, cancer cells exploit glycogen metabolism for optimal glucose utilization. Different *in vitro* studies have demonstrated glycogen accumulation without inhibiting or interfering with the breakdown process (Favaro et al., 2012; Pelletier et al., 2012).

Glycogen accumulation and breakdown have been variably observed under hypoxic conditions. The relative rates of those opposing pathways changes with the chronicity of hypoxia, where there is early glycogen accumulation followed by a gradual decline (Favaro et al., 2012). GYS1 induction (and therefore glycogen accumulation) is dependent on HIF1α, a central factor in the response to hypoxia. Indeed, M2 tumors may be a result of a hypoxic microenvironment. Using previously validated gene panels on bulk samples, hypoxia scores were higher in M2 tumors. Consistent with this, angiogenesis-related genes were upregulated. On the other hand, specifically in the tumor compartment, only 32 of 212 hypoxia related genes were dysregulated. This suggests that changes in hypoxia-related genes mostly resided in the stromal compartment, which is consistent with previous report (Mello et al., 2022).

Finally, the potential role of the renin-angiotensin in M2 tumors was interesting. The local RAS system encourages tumor cell proliferation and angiogenesis by upregulating EGFR and VEGF expression (Anandanadesan et al., 2007) and RAS inhibition induces apoptosis in pancreatic cancer cells (Gong et al., 2010). In a murine model of PDAC, gemcitabine and the angiotensin receptor blocker (ARB) losartan synergistically inhibit tumor growth via VEGF suppression (Noguchi et al., 2009). There have been clinical reports of survival benefits in PDAC patients receiving RAS inhibitors who were treated with gemcitabine (Nakai et al., 2010) and with resection (Cerullo et al., 2017). A large retrospective study of 8,158 PDAC patients reported survival benefits related to RAS inhibition, more pronounced with ARBs than angiotensin I converting enzyme (ACE) inhibitors (Keith et al., 2022). Further studies will be required to determine whether our metabolic subtypes confer differential sensitivity to RAS inhibition.

In conclusion, we have described two metabolic subtypes based on gene expression in tumor cells. Our unbiased approach in identifying the clinically most impactful genes unveiled novel phenotypes. We are unable to discern whether there are host factors that contribute to the metabolic phenotypes. The incidence of diabetes was similar in patients with M1 and M2 tumors, but BMI data were lacking. Race distribution was similar in patients with M1 and M2 tumors, but the majority of patients were white. Further studies in patients who are not white are warranted. We have not yet identified how our tumor classification is relevant to therapeutic decisions. Indeed, none of the molecular subtypes described for PDAC have yet proven relevant to clinical decision-making. However, some interesting biological phenomena have been uncovered that warrant further investigation. Geospatial studies would be particularly informative in this regard.

## Data Availability

Publicly available datasets were analyzed in this study. This data can be found here: https://portal.gdc.cancer.gov, https://dcc.icgc.org, https://ega-archive.org/studies/EGAS00001002543, https://www.orcestra.ca, https://www.ncbi.nlm.nih.gov/geo/query/acc.cgi?acc=GSE154778.

## Supplementary material

SUPPLEMENTARY FIGURE S1 Visualization of predicted metabolic subtypes in all validation cohorts. The heatmaps exhibit the expression of 26 prognostic metabolic genes across the identified metabolic subtypes within each validation cohort. The proportion of patients belong to each subgroup are shown in the figures. The gene order follows the same order as depicted in the discovery cohort. Expression of identified prognostic metabolic genes are similar to the discovery set and highlight noticeable differences between groups.

SUPPLEMENTARY FIGURE S2 Glycogen metabolism related gene expression in identified metabolic subtypes. **(A)** Gene set enrichment analysis focussed on glycogen metabolism, showing enrichment of glycogen catabolism in the M2 phenotype accompanied by enrichment of glycogen synthesis pathways. **(B)** Scatter plot shows expression levels of regulators involved in glycogen synthesis and degradation process. Mann- Whitney U tests p values are shown at the top of the plots.

SUPPLEMENTARY FIGURE S3 Tumor cell composition in metabolic subtypes. Three different methods were applied to estimate the proportion of tumor that is comprised of tumor cells: Absolute purity, DeMixT and tumor nuclei counts. Violin plots show higher tumor content in the M2 phenotype and the differences in tumor content are significantly different between M1 and M2 based on *p*-value <0.05.

SUPPLEMENTARY FIGURE S4 Expression of prognostic metabolic genes at a single cell level. Single cell data were used to determine the expression of prognostic genes in malignant epithelial cells. Each heatmap represents gene expression of prognostic metabolic genes in a single tumor. The x-axis depicts data from individual epithelial tumor cells, and the y-axis depicts data from the 26 prognostic metabolic genes.

SUPPLEMENTARY FIGURE S5 Predicted metabolic phenotypes in PDAC cell lines. The heatmap shows two distinct groups based on the 26 prognostic metabolic gene expressions in individual PDAC cell line cohorts (CCLE and gCSI). In each cell line collection, the proportion of cells with each metabolic subtype are similar.

SUPPLEMENTARY FIGURE S6 Drug target analysis of PDAC cell lines. Sensitivity to antineoplastic drugs was assessed in cell lines identified as M1 and M2 cell lines. Each box plot shows area above the drug-response curve (AAC) values for drug screening in CCLE and gCSI cohorts, respectively. The p-values based on the Wilcoxon test are indicated on the top of box plots.

SUPPLEMENTARY TABLE S1 Prognostic metabolic genes, and summary of functions.

SUPPLEMENTARY DATA SHEET S1 Prognostic metabolic genes and differentially expressed genes list.
