# Supplementary figures and images for: Clinically impactful metabolic subtypes of pancreatic ductal adenocarcinoma (PDAC)
Source: Front Genet. 2023 Oct 30;14:1282824. doi: 10.3389/fgene.2023.1282824 (PMC10643182; doi:10.3389/fgene.2023.1282824)

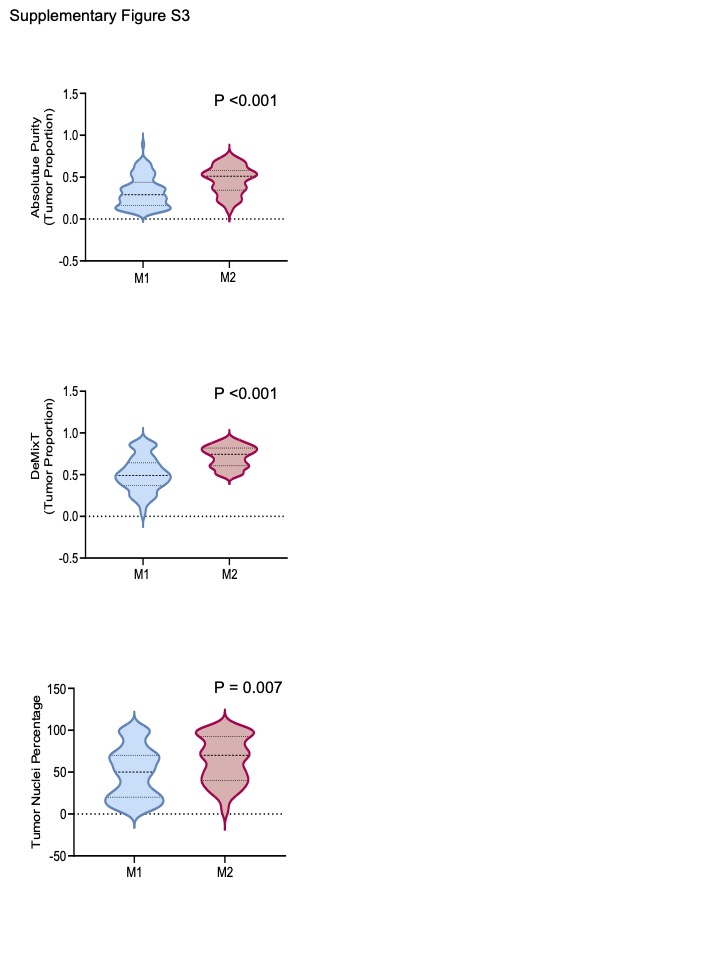

Supplement: Supplementary file 1 [file Image3.jpeg]

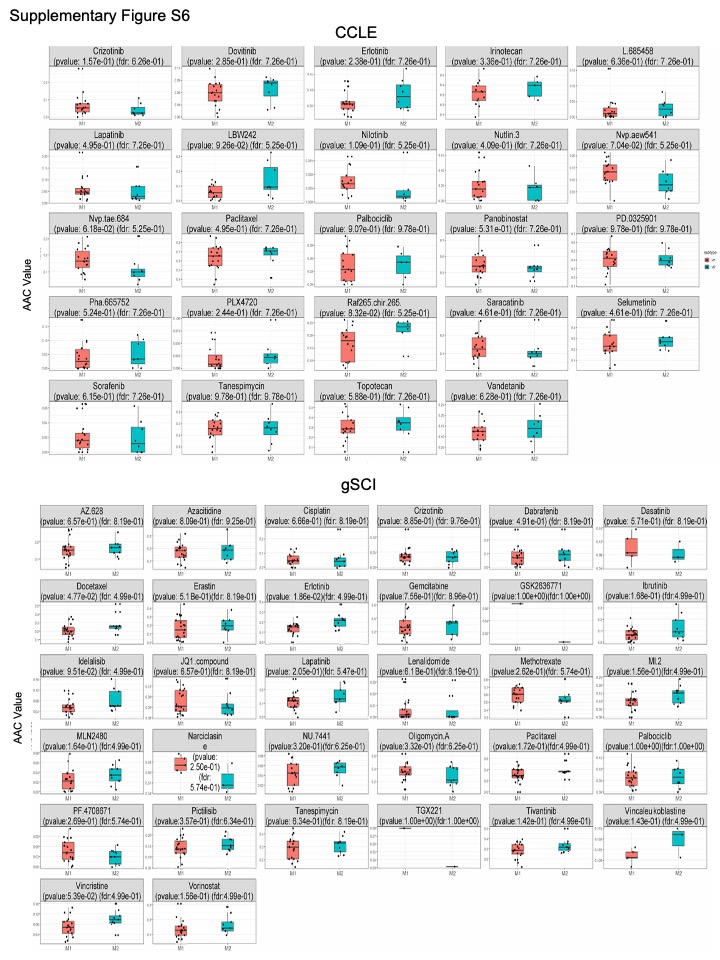

Supplement: Supplementary file 2 [file Image6.jpg]

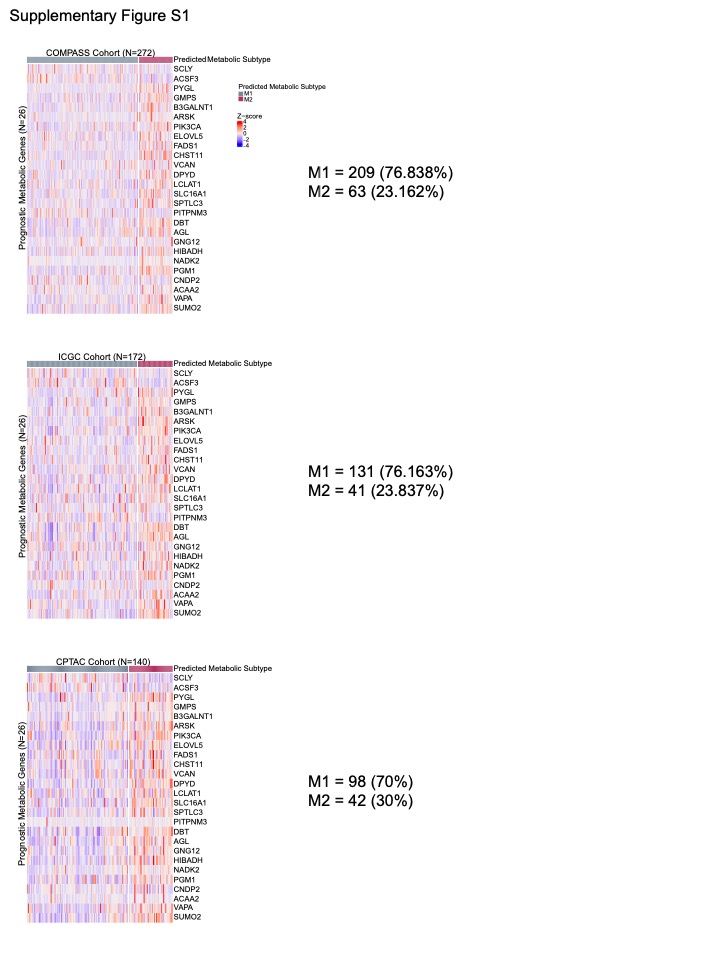

Supplement: Supplementary file 4 [file Image1.jpeg]

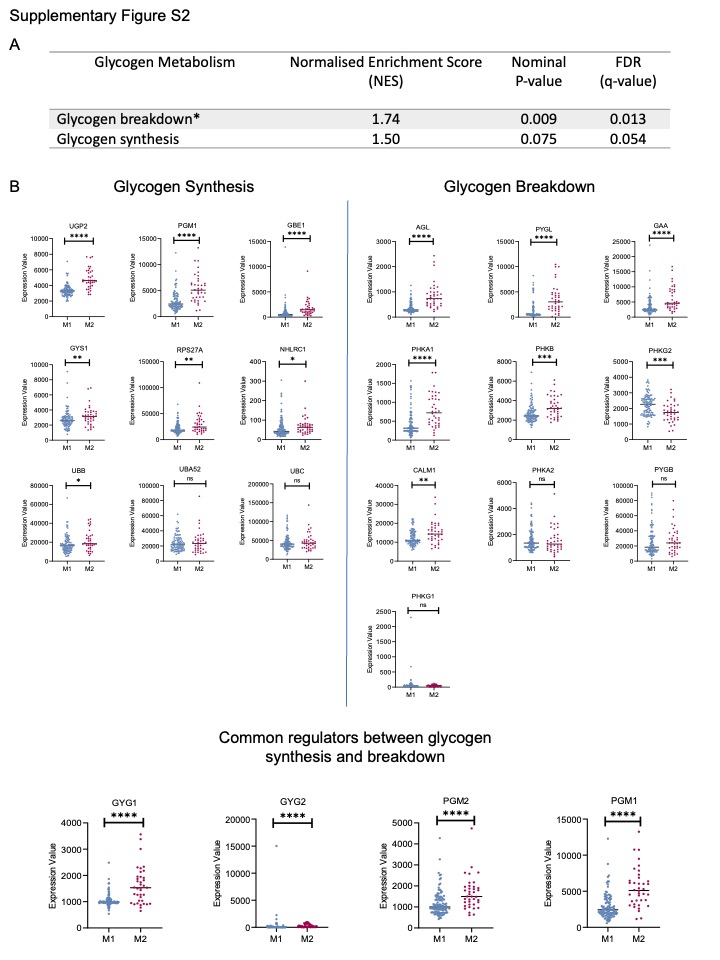

Supplement: Supplementary file 5 [file Image2.jpeg]

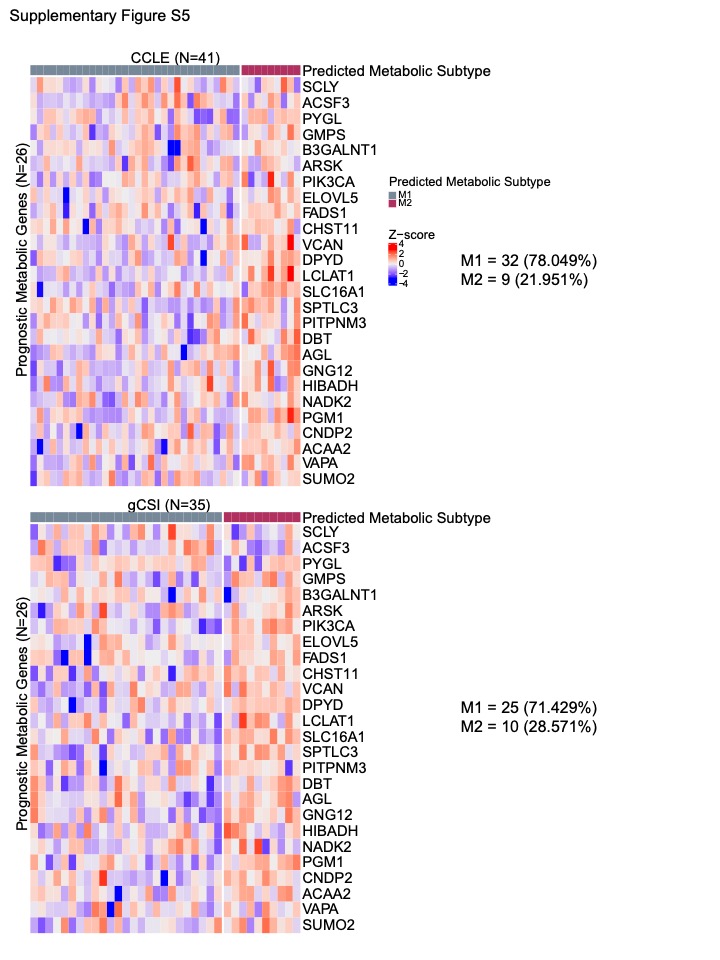

Supplement: Supplementary file 6 [file Image5.jpeg]

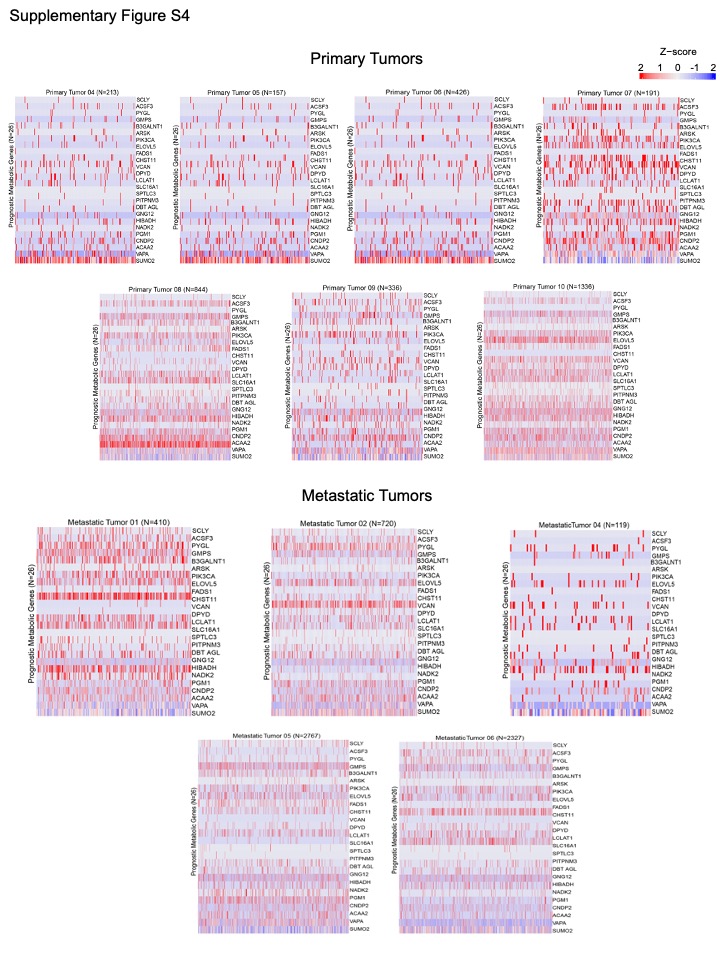

Supplement: Supplementary file 7 [file Image4.jpg]
