# Supplementary material for: Clinically impactful metabolic subtypes of pancreatic ductal adenocarcinoma (PDAC)
Source: Front Genet. 2023 Oct 30;14:1282824. doi: 10.3389/fgene.2023.1282824 (PMC10643182; doi:10.3389/fgene.2023.1282824)
Supplement: Supplementary file 3 [file Table1.docx]

| **Prognostic Metabolic Genes** | **Cox-coefficient** | **Wald**  **P-value** | **Summary of Functions** |
| --- | --- | --- | --- |
| DPYD  (Dihydropyrimidine Dehydrogenase) | 0.47 | 6.46E-04 | Involved in degradation of pyrimidine and chemotherapeutic drug 5-fluraoracil. |
| LCLAT1  (Lysocardiolipin Acyltransferase 1) | 0.57 | 4.46E-04 | Function as an acyltransferase in endoplasmic reticulum (ER) and involved in phosphatidylinositol and phosphatidylglycerol remodeling. |
| SPTLC3  (Serine Palmitoyl transferase Long Chain Base) | 0.55 | 4.84E-04 | Involved in sphingolipid biosynthesis. Prognostic marker in renal cancer. |
| DBT  (Dihydrolipoamide Branched Chain Transacylse) | 0.50 | 6.01E-04 | Involved in breakdown of branched-chain amino acids isoleucine, leucine, and valine. Prognostic marker in renal cancer. |
| HIBADH  (3-Hydroxyisobutyrate Dehydrogenase) | 0.41 | 4.76E-03 | Involved in branched-chain amino acid catabolism. |
| NADK2  (NAD Kinase 2) | 0.52 | 4.96E-04 | Involved in phosphorylation of NAD to produce NADP. |
| PYGL  (Glycogen  Phosphorylase L) | 0.48 | 5.05E-04 | Involved in glycogen and carbohydrate metabolism, converts phosphorolytic cleavage of glycogen to glucose-1-phosphate. |
| SCLY  (Selenocysteine Lyase) | -0.58 | 5.48E-04 | Involved in selenoprotein biosynthesis; catalyzes the decomposition of L-selenocysteine to L-alanine and elemental selenium. |
| GMPS  (Guanine Monophosphate Synthase) | 0.64 | 4.97E-05 | Involved in de novo synthesis of purine nucleotides (guanine). Unfavorable prognostic marker in liver, pancreatic and endometrial cancer. |
| CNDP2  (Carnosine Dipeptidase 2) | 0.32 | 2.47E-02 | Catalyzed the production of N-lactoyl-amino-acids from lactate and amino acids by reverse proteolysis. |
| ELOVL5  (ELOVL Fatty Acid Elongase 5) | 0.47 | 6.46E-04 | Involved in fatty-acid and lipid metabolism. It catalyzes first and rate-limiting reaction of the four reaction that constitute the long-chain fatty-acid elongation cycle. |
| GNG12  (Guanine nucleotide-binding protein Subunit Gamma 12) | 0.34 | 2.17E-02 | Involved in various transmembrane signaling processes including GTPase activity, replacement of GDP to GTP. |
| AGL  (Glycogen Debranching Enzyme) | 0.42 | 5.45E-03 | Involved in glycogen synthesis; it provides instruction for making the glycogen debranching enzyme. |
| VAPA  (VAMP Associated  Protein A) | 0.37 | 3.04E-03 | Involved in sphingolipid metabolism. Functions including membrane fusion, protein complex assembly and cell motility. |
| FADS1  (Fatty Acid Desaturase 1) | 0.38 | 2.75E-03 | Involved in fatty acid biosynthesis, lipid metabolism. It controls fatty acid unsaturation by forming double bonds between certain carbons in the acyl chain. |
| ACAA2  (ACETYL-CoA Acyltransferase 2) | 0.41 | 4.09E-03 | Involved in fatty acid metabolism; catalyzes the last step of fatty acid beta-oxidation process (in mitochondria). |
| PIK3CA  (Phosphoinositide 3-kinase Alpha) | 0.40 | 4.25E-03 | Known as oncogene. Involved in phosphatidylinositol metabolism and angiogenesis. Mutant PIK3CA also plays a critical role in altered glutamine metabolism. |
| CHST11  (Carbohydrate Sulfotransferase 11) | 0.43 | 3.59E-03 | Involved in carbohydrate metabolism. CHST11 negatively modulates metastasis and drug resistance in hepatocellular carcinoma. |
| SUMO2 (Small Ubiquitin Like Modifier 2) | 0.40 | 1.02E-02 | Involved in facilitating cellular stress responses via energy and nucleotide metabolism. |
| ACSF3  (Acyl-CoA Synthetase Family Member 3) | -0.46 | 8.47E-03 | Involved in fatty acid metabolism by catalyzing of a thioester linkage between fatty acids and coenzyme A. Also, a favorable prognostic marker of pancreatic cancer. |
| SLC16A1  (Solute Carrier Family 16 Member 1) | 0.35 | 2.11E-02 | Involved in the movement of lactate and pyruvate (monocarboxylates) across the membrane. |
| B3GALNT1  (Globoside Synthase) | 0.53 | 3.79E-04 | Involved in sphingolipid metabolism; it transfers N-acetyl galactosamine onto globotriaosylceremide. |
| PGM1  (Phosphoglucomutase 1) | 0.37 | 3.36E-03 | Involved in both breaking down and synthesis of glucose. |
| ARSK  (Arylsulfatase Family Member K) | 0.45 | 2.19E-03 | Involved in hormone biosynthesis, sphingolipid metabolism and metabolism of proteins. It hydrolyzes sulfate easters from sulfated carbohydrates, steroids and glycolipids. |
| PITPNM3  (Atypical Chemokine Receptor 6) | 0.42 | 3.33E-03 | Involved in the transfer of phosphatidylinositol and phosphatidylcholine between membranes. |
| VCAN  (Versican) | 0.45 | 3.90E-03 | Involved in intracellular signaling and cell adhesion. It facilitates the assembly of the extracellular matrix and maintains its stability. |
